# Acute cardiac tamponade during atrial flutter ablation: improved hemodynamics after positive pressure ventilation: a case report

**DOI:** 10.1186/s13256-023-04268-3

**Published:** 2023-12-21

**Authors:** Roger L. Royster, Scott R. Coleman, Eduardo J. Goenaga-Díaz, Karl M. Richardson, S. Patrick Whalen

**Affiliations:** 1 Department of Anesthesiology, Wake Forest University School of Medicine, Medical Center Boulevard, Winston-Salem, NC 27157-1009 USA; 2 Department of Cardiology, Wake Forest University School of Medicine, Winston-Salem, NC USA; 3 Department of Anesthesiology and Critical Care Medicine, Children’s Hospital of Philadelphia, Philadelphia, PA USA

**Keywords:** Atrial flutter ablation, Cardiac resuscitation, Cardiac tamponade, Catecholamines, Mode of ventilation

## Abstract

**Introduction:**

Acute cardiac tamponade is a rare event during any type of interventional or surgical procedure. It can occur during electrophysiology procedures due to radiofrequency ablation, lead or catheter manipulation, transseptal puncture, laser lead extractions, or left atrial appendage occlusion device positioning. Cardiac tamponade is difficult to study in a prospective manner, and case reports and case series are important contributions to understanding the best options for patient care.

**Case summary:**

An 87-year-old Caucasian male patient breathing spontaneously developed acute tamponade during an atrial flutter ablation. Pericardial drain insertion was difficult, and hypotension failed to respond to epinephrine boluses. The patient became hypoxemic and hypercarbic, requiring intubation. Unexpectedly, the blood pressure markedly increased postintubation and remained in a normal range until the pericardium was drained.

**Conclusion:**

Spontaneous ventilation is considered important to maintain venous return to the right heart during cardiac tamponade. However, spontaneous ventilation reduces venous return to the left heart and worsens the paradoxical pulse in tamponade. Intravenous vasopressors are thought to be ineffective during cardiac tamponade. Our patient maintained pulmonary blood flow as indicated by end-tidal carbon dioxide measurements but had no measurable systemic blood pressure during spontaneous ventilation. Our case demonstrates that tracheal intubation and positive pressure ventilation can transiently improve left heart venous return, systemic perfusion, and drug delivery to the systemic circulation.

## Background

Cardiac tamponade is a rare complication during electrophysiology procedures, occurring in less than 1% of patients. Tamponade occurs in lead or catheter manipulation, in procedures requiring transseptal puncture, in laser lead extractions, and in left atrial appendage occlusion device positioning. A steam pop is an audible sound that occurs during radiofrequency ablation resulting from interstitial fluid heating to as high as 100 °C and forming a gas that causes an intramyocardial explosion, which can perforate the heart.

There are few patient studies of cardiac tamponade with most clinical information resulting from case reports or case series [1]. Research on tamponade performed in animals requiring instrumentation of various cardiothoracic structures may or may not mimic the physiology of tamponade in humans [2].

Cardiopulmonary interactions in tamponade are related to the impact of spontaneous ventilation (SV) and positive pressure ventilation (PPV) [3]. We report an unusual case of steam pop-induced cardiac tamponade that hemodynamically improved after intubation and PPV prior to the drainage of pericardial blood.

## Case presentation

An 87-year-old Caucasian male patient with history of typical atrial flutter was scheduled for a right heart atrial flutter ablation. Preoperative electrocardiogram showed typical atrial flutter with negative flutter waves in leads 2, 3, and aVF, positive flutter waves in V1, and a ventricular rate of 144 beats per minute (bpm). Preoperative transesophageal echocardiogram (TEE) revealed no clot in the left atrial appendage, mildly reduced left ventricular function, and no pericardial effusion.

The patient received intravenous propofol titrated to 65 μg/kg/minute, breathing spontaneously with mask delivery of 60% oxygen. End-tidal carbon dioxide (EtCO_2_) was measured continuously. The blood pressure (BP) via cuff was 116/66 mmHg. Radiofrequency ablation was performed using an open irrigation, contact force-monitoring catheter delivering 30–35 watts of power.

**Fig. 1:**
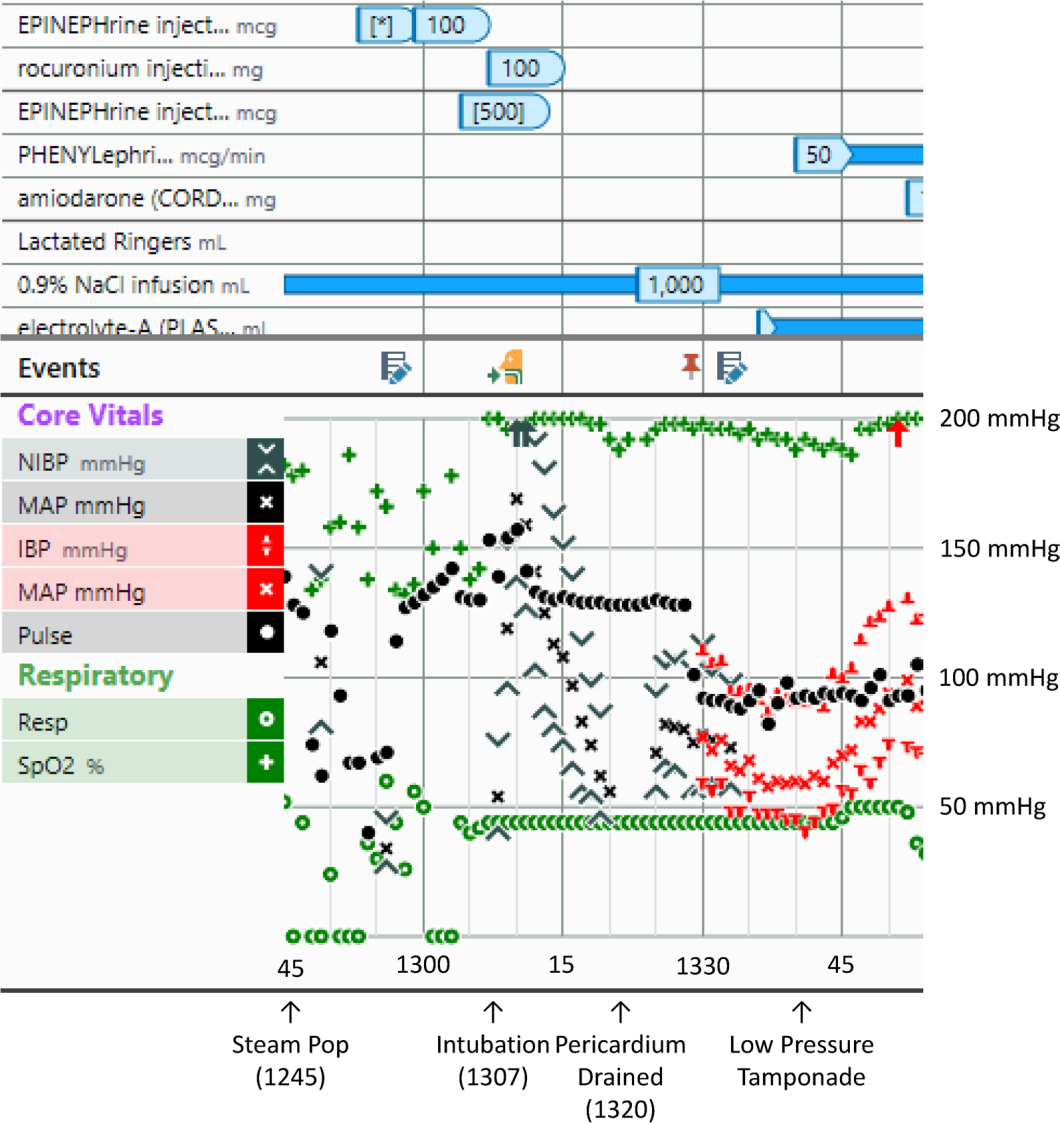
Electronic anesthesia record of hemodynamic events during pericardial tamponade secondary to ablation of atrial flutter demonstrating the marked increase in blood pressure minutes after intubation and positive pressure ventilation

During a 7-second ablation at 35 watts, the attending cardiologist felt a sudden resistance in his ablation catheter and a steam pop was noted. Over the next few minutes, the BP became unmeasurable, and 100 μg of intravenous (IV) epinephrine was administered twice over several minutes (Fig. 1). Propofol was discontinued. The cardiologist performed a transthoracic echocardiogram (TTE) that showed a large pericardial effusion and began preparation for a pericardial drain. Blood pressure was measured as 44/29 mmHg, and an additional 100 μg of epinephrine was administered (Table 1). The pericardium was punctured, and a guidewire and catheter were inserted; however, positioning the catheter to drain the pericardium was difficult. The patient had continuous measurements of EtCO_2_ but without a BP measurement. The cardiologist reported a weak femoral pulse. Another 200 μg of epinephrine was administered and, several minutes later, 300 μg of epinephrine. At this time, the patient was receiving 100% oxygen, but the pulse oximetry saturation had decreased to 70% with an EtCO_2_ of 59 mmHg. The patient was administered 100 mg of rocuronium and intubated. The BP postintubation was measured at 74/42 mmHg and, surprisingly, slowly increased to 190/105 mmHg and remained in a normal range until 600 ml of blood was drained from the pericardium; the BP stabilized at 100/70 mmHg.

**Table 1:** Timeline of events*

Time (hours)	Event
1245	Steam pop
1249	BP 139/83 mmHg
1253	100 µg epinephrine
1255	BP 44/29 mmHg
1256	Pericardial effusion seen on TTE
1257	100 µg epinephrine
1302	Pericardium puncture
1304	Pericardial catheter inserted, 200 µg epinephrine
1306	Unable to drain blood, 300 µg epinephrine, Sat% 71, EtCO_2_ 59
1307	Intubation
1308	BP 74/42 mmHg, Sat% 100, EtCO_2_ 38
1309	BP 150/98 mmHg
1312	BP 190/105 mmHg
1319	BP 85/48 mmHg
1320	600 ml blood drained from pericardium, BP 100/70 mmHg
1330	Femoral arterial line inserted, TEE probe inserted
1338	BP 95/45 mmHg, 400 ml plasmalyte
1350	BP 128/74 mmHg

*Times taken from electronic medical record and records of electrophysiology lab

*BP* blood pressure, *TTE* transthoracic echocardiograph, *EtCO*_*2*_ end-tidal carbon dioxide, *Sat%* saturation percentage, *TEE* transesophageal echocardiography

After pericardial drainage, an arterial catheter was placed in the femoral artery and a TEE probe was inserted, which revealed reduced heart volumes and a small amount of blood in the pericardium with right ventricular diastolic collapse. Low pressure tamponade was diagnosed and crystalloid (500 ml) was administered; the BP increased to 125/70 mmHg. The patient required one further pericardial drainage because of recurrent tamponade diagnosed by transthoracic echocardiogram (TTE) in the intensive care unit and was discharged from the hospital in several days after repeat TTEs showed no pericardial effusion.

## Discussion and conclusion

Kussmaul described the arterial pulse “going away” during inspiration and coined the term “paradoxical pulse” [4]. This was the first description in which SV inhibited left ventricular stroke volume by reducing left ventricular venous return. Multiple studies since have demonstrated that inspiration reduces left ventricular stroke volume under normal physiologic conditions with a reduction of systolic BP of less than 6 mmHg [5].

**Fig. 2:**
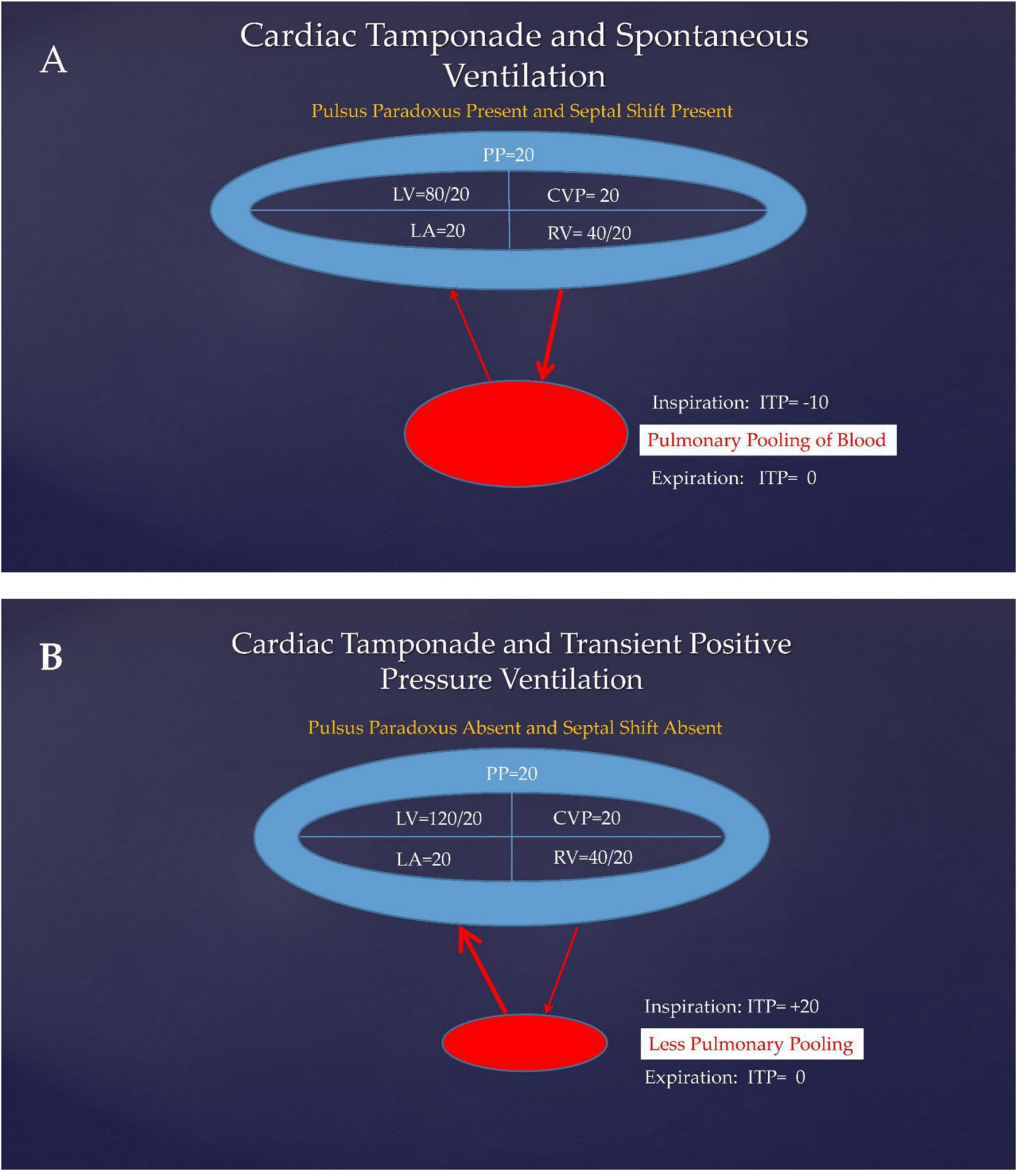
Diagram illustrating cardiac systolic and diastolic pressures and pericardial pressure during global tamponade, relative changes in pulmonary arterial and pulmonary venous blood flow, and pulmonary pooling of blood in **A** during spontaneous ventilation and in **B** during positive pressure ventilation. *PP* pericardial pressure, *CVP* central venous pressure, *RV* right ventricle, *LA* left atrium, *LV* left ventricle, *PA* pulmonary artery, *PV* pulmonary vein, *ITP* intrathoracic pressure. All pressures in mmHg

Spontaneous ventilation normally augments right heart venous return by creating negative intrathoracic pressure. During tamponade, because of increased ventricular interdependence caused by pericardial constraint, a “downhill” pressure gradient develops between the high pressure extracardiac venous system and the low pressure pulmonary circulation. Echocardiograms show that inspiration causes a significantly greater increase in venous return during tamponade, demonstrated by increased tricuspid valve in-flow velocities and a greater reduction in left heart venous return seen by reduced mitral valve in-flow velocities (Table 2) [6]. The venous return to the left heart is inhibited because of the “uphill” pressure gradient moving blood from the low-pressure pulmonary circulation into the tamponaded left heart (Fig. 2A). This results in an interventricular septal shift to the left and an increased paradoxical pulse with a reduction in systolic BP during inspiration of greater than 10 mmHg.

**Table 2:** Impact of spontaneous and positive pressure ventilation on mitral and tricuspid valve peak flow velocities and paradoxical pulse with and without cardiac tamponade

	MV	TV	PP
Spontaneous ventilation without tamponade			
Inspiration	↓ < 10%	↑	< 6 mmHg
Expiration	↑	↓ < 25%	
Spontaneous ventilation with tamponade			
Inspiration	↓↓ 25–65%	↑↑ 58–85%	> 10 mmHg
Expiration	↑↑ 22%	↓↓	
Positive pressure ventilation without tamponade			
Inspiration	↑	↓	Reverse paradox
Expiration	↓	↑	
Positive pressure ventilation with tamponade			
Inspiration	↑	↓	Absent
Expiration	↓	↑	

*MV* mitral valve, *TV* tricuspid valve, *PP* paradoxical pulse

In our case, despite maintaining good pulmonary blood flow indicated by good EtCO_2_ measurements with SV, systemic hypotension developed. Blood tends to pool in the lungs with a resultant increase in extravascular lung water because of the reduced left heart venous return [7, 8]. However, with cardiac compressive syndromes such as tamponade—even with intracardiac pressures equal to pressures in congestive heart failure—alveolar edema often does not develop [9]. Some hypothesize that natriuretic peptide secretion is not increased in compressive syndromes as compared with disease states that stretch the heart, and this has an impact on capillary permeability [9]. The pulmonary pooling of blood and negative impact on left heart diastolic filling may explain why intravenous vasopressors are often ineffective during tamponade [10]. Additionally, chest compressions were not performed in our case and may not be effective in tamponade [11].

In animal studies of cardiac tamponade, PPV reduces venous return to the right ventricle but improves left heart filling and systemic pressures [3]. The paradoxical pulse in tamponade has been described in case reports in humans to be reduced, to disappear, or to be reversed with PPV [12]. This reduction of ventricular interdependence and the resultant loss of the paradoxical pulse suggests improved venous return from the lungs to the left ventricle that increases systemic blood flow, pressures (Fig. 2B), and catecholamine delivery.

There are case reports of patients with tamponade who need intubation for respiratory support, pericardiocentesis, or pericardial windows and do well hemodynamically. Data from 105 patients undergoing pericardial window for tamponade indicate that the outcome is no different between patients managed with local anesthesia and sedation and patients receiving general anesthesia and intubation, although more vasopressor administration was required during general anesthesia [13]. However, prolonged PPV without pericardial drainage may eventually have a detrimental effect on right heart venous return and hemodynamics. Elimination of positive end expiratory pressure and reduction in tidal volumes with increased respiratory rates during mechanical ventilation may help reduce the negative impact of PPV [14].

After pericardial drainage, the BP remained marginal and a TEE demonstrated a small amount of pericardial blood causing right ventricular diastolic collapse indicating low-pressure tamponade. This results when pericardial pressure becomes greater than right atrial or right ventricular end diastolic pressure in patients who are hypovolemic. Tamponade can occur in patients and animals at pericardial pressures less than 5 mmHg [4]. The maintenance of SV is more important in patients with low-pressure tamponade to maintain right ventricular volumes and pressures. Administration of fluid can increase right heart pressures above pericardial pressure and markedly improve systemic pressures in low-pressure tamponade with systolic BP less than 100 mmHg being the best predictive factor [15].

This case demonstrates a patient in cardiac tamponade whose systemic perfusion improved with intubation and PPV prior to pericardial drainage. This suggested improved left ventricular filling and increased perfusion and drug delivery to the systemic circulation. PPV in patients with worsening respiratory distress should not be avoided, especially when pericardial drainage is eminent. These are important physiologic points for physicians to consider when trying to make the right decision about how to manage these acutely ill, often unstable, patients.

## Data Availability

Data sharing is not applicable to this article as no datasets were generated or analyzed during the current study.

## Abbreviations

BP: Blood pressure
EtCO_2_: End-tidal carbon dioxide
PPV: Positive pressure ventilation
SV: Spontaneous ventilation
TEE: Transesophageal echocardiography
TTE: Transthoracic echocardiography

## References

[1] White JB, Macklin S, Studley JG, Marshall RD (1988). Cardiac tamponade: a review of diagnosis and anaesthetic and surgical management illustrated by three case reports. Ann R Coll Surg Engl.

[2] Faehnrich JA, Noone RB, White WD, Leone BJ, Hilton AK, Sreeram GM (2003). Effects of positive-pressure ventilation, pericardial effusion, and cardiac tamponade on respiratory variation in transmitral flow velocities. J Cardiothorac Vasc Anesth.

[3] Möller CT, Schoonbee CG, Rosendorff C (1979). Haemodynamics of cardiac tamponade during various modes of ventilation. Br J Anaesth.

[4] Bilchick KC, Wise RA (2002). Paradoxical physical findings described by Kussmaul: pulsus paradoxus and Kussmaul’s sign. Lancet.

[5] Claessen G, Claus P, Delcroix M, Bogaert J, La Gerche A, Heidbuchel H (2014). Interaction between respiration and right versus left ventricular volumes at rest and during exercise: a real-time cardiac magnetic resonance study. Am J Physiol Heart Circ Physiol.

[6] Appleton CP, Hatle LK, Popp RL (1988). Cardiac tamponade and pericardial effusion: respiratory variation in transvalvular flow velocities studied by Doppler echocardiography. J Am Coll Cardiol.

[7] Sznajder JI, Evander E, Pollak ER, Becker C, Little AG (1987). Pericardial effusion causes interstitial pulmonary edema in dogs. Circulation.

[8] Kitashiro S, Sugiura T, Tamura T, Izuoka T, Miyoshi H, Saito D (1999). Factors associated with increased extravascular lung water in cardiac tamponade and myocardial ischemia. Crit Care Med.

[9] Spodick DH (1992). Cardiogenic pulmonary edema and its absence in cardiac tamponade and constriction. A role for atrial natriuretic factor?. Chest.

[10] Kearns MJ, Walley KR (2018). Tamponade: hemodynamic and echocardiographic diagnosis. Chest.

[11] Luna GK, Pavlin EG, Kirkman T, Copass MK, Rice CL (1989). Hemodynamic effects of external cardiac massage in trauma shock. J Trauma.

[12] Michard F (2005). Changes in arterial pressure during mechanical ventilation. Anesthesiology.

[13] O'Connor CJ, Tuman KJ (2010). The intraoperative management of patients with pericardial tamponade. Anesthesiol Clin.

[14] Mattila I, Takkunen O, Mattila P, Harjula A, Mattila S, Merikallio E (1984). Cardiac tamponade and different modes of artificial ventilation. Acta Anaesthesiol Scand.

[15] Sagristà-Sauleda J, Angel J, Sambola A, Permanyer-Miralda G (2008). Hemodynamic effects of volume expansion in patients with cardiac tamponade. Circulation.

